# Supplementary material for: The soft tissue and skeletal anatomy of two Late Jurassic ichthyosaur specimens from the Solnhofen archipelago
Source: PeerJ. 2022 Apr 7;10:e13173. doi: 10.7717/peerj.13173 (PMC8995021; doi:10.7717/peerj.13173)
Supplement: Supplemental Information 1 [file peerj-10-13173-s001.docx]

Delsett et al.: The Skeletal anatomy and Soft Tissue of two Late Jurassic Ichthyosaur Specimens from the Solnhofen Archipelago

SUPPLEMENTARY INFORMATION. Apatite calculations

In order to evaluate the possibility of soft tissue as the P source for phosphatization, a mass balance was carried out. As ichthyosaurs have no living descendants, we used whales for our calculations, as dolphins and porpoises share body shape with derived ichthyosaurs in order to reduce drag (Massare, 1988), and both were endothermic pursuit predators (Bernard et al., 2010). We base our calculations on data reported for river dolphins (*Pontoporia blainvillei*) and for P content in organs from bowhead whale (O'Hara et al., 2004; Caon et al., 2007).

**Abbreviations**

c: concentration

FAP: fluorapatite – Ca_5_(PO_4_)_3_F

M: molar mass (g/mol)

m: mass

n: moles

ρ: density (g/cm^3^)

V: volume (cm^3^)

**Chemical equations**

ρ = m/V

n = m/M

**Constants used**

M (P): 30.974 g/mol

M (FAP): 504.30 g/mol

ρ (FAP): 3.2 g/cm^3^

c (P in blubber): 7.43 mg/100 g = 0.0743 mg/g (O’Hara et al., 2004)

c (P in skin): 192.3 mg/100 g = 1.923 mg/g (O’Hara et al., 2004)

c (P in muscular tissues): 205.5 mg/100 g = 2.055 mg/g (O’Hara et al., 2004)

**Determining weight and soft tissue content**

From Caon et al. (2007) it is possible to calculate the total weight (TW) based on body length (L) using the equation below. Our specimen has been measured to a total length of 161 cm.

TW: 0.4289 x L – 27.715 = 0.4289 x 161 – 27.715 = 41.3 Kg.

For the purpose of this paper we make the assumption that blubber plus muscles are 50% of the total weight i.e. ignoring intestine and skeletal elements. Consequently, the total weight of muscle and blubber is 20.67 Kg.

Caon et al. (2007) found the following correlation between body length (L) and blubber weight percentages (BW%):

BW% = -0.1305 x L + 44.114.

Applying the above equation we get

BW% = -0.1305 x 161 + 44.114 = 23.10 %

We can now calculate the total weight of blubber and muscular tissue as follows

Blubber = 20.67 kg x 23.10 / 100 = 4.78 Kg = 4780 g

Muscular tissue = TW – Blubber = 20.67 Kg – 4.78 Kg = 15.89 Kg = 15890 g

It has not been possible to find literature on the phosphorus content in *Pontoporia blainvillei* soft tissue. Therefore, we have used the detailed information for bowhead whales presented by O’Hara et al. (2004). We use the average P concentration (P ave.) from skin and muscular tissue.

c (P ave.) = (1.923 mg/g + 2.055 mg/g) / 2 = 1.989 mg/g.

Calculation of total amount of phosphorus in blubber and other tissue.

m (P in Blubber)=m (Blubber) x c (P in Blubber) = 4780 g x 0.0743 mg/g = 355.15 mg = 0.355 g

m (P in other tissue) = 15890 g x 1.989 mg/g = 31605 mg = 31.605 g

m (P total) = 0.355 g + 31.605 g = 31.96 g

**Calculations on how much fluorapatite can be formed**

The combined phosphorus content in skin, muscular tissue and blubber is therefore 31.96 g. Based on this it is possible to apply simple chemical equations to determine how much fluorapatite that can be formed.

n (P) = m (P)/M (P) = 31.96 g / 30.974 g/mol = 1.03 mol

From the formula of fluorapatite it is seen that the molar ratio between apatite and P is 1:3, i.e. it requires three moles of P to form one mole of fluorapatite. We can now determine how much fluorapatite we can form:

n (FAP) = n (P) /3 = 1.03 mol / 3 = 0.344 mol

m (FAP) = n (FAP) x M (FAP) = 0.344 mol x 504.30 g/mol = 173.45 g

V (FAP) = m (FAP) / ρ (FAP) = 173.45 g / 3.2 g/cm^3^ = 54.20 cm^3^

That means the P released from the animal can form 173.45 g fluorapatite, which, if it was a solid crystal, would have a volume of 54.20 cm^3^

Based on image analyses of Fig. 2a and the body length of 161 cm, we have calculated the specimen to cover an area of 6560 cm^2^. From the volume of formed apatite we can calculate how thick a layer of apatite that could form under the fossil, assuming uniform distribution over the entire area.

Thickness of fluorapatite = 54.20 cm^3^ / 6560 cm^2^ = 0.00826 cm = 0.826 mm

LITERATURE CITED

Bernard, A., C. Lécuyer, P. Vincent, R. Amiot, N. Bardet, E. Buffetaut, G. Cuny, F. Fourel, F. Martineau, J.-M. Mazin, and A. Prieur. 2010. Regulation of body temperature by some Mesozoic marine reptiles. Science 328:1379-1382.

Caon, G., C. B. Fialho, and D. Danilewicz. 2007. Body fat condition in franciscanas (*Pontoporia blainvillei)* in Rio Grande do Sul, Southern Brazil. Journal of Mammalogy 88:1335–1341.

Massare, J. A. 1988. Swimming capabilities of Mesozoic marine reptiles: implications for methods of predation. Paleobiology 14:187-205.

O'Hara, T. M., P. Hoekstra, C. Hanns, D. Muir, D. Wetzel, and J. Reynolds. 2004. A preliminary assessment of the nutritive value of select tissues from the bowhead whale based on suggested nutrient daily intakes. International Whaling Commission, Scientific Committee Report, SC/56/E2.
